# Bacillary Angiomatosis and Bacteremia due to *Bartonella quintana* in a Patient with Chronic Lymphocytic Leukemia

**DOI:** 10.1155/2013/694765

**Published:** 2013-04-28

**Authors:** Rosamaria Fulchini, Guido Bloemberg, Katia Boggian

**Affiliations:** ^1^Division of Infectious Diseases, Department of Internal Medicine, Cantonal Hospital of St. Gallen, 9007 St. Gallen, Switzerland; ^2^Institute of Medical Microbiology, University of Zurich, 8006 Zurich, Switzerland

## Abstract

We present a 63-year-old man treated with alemtuzumab for chronic lymphocytic leukemia who developed multiple angiomatous papules and fever. Real-time polymerase chain reaction (RT-PCR) from a skin lesion and blood sample revealed *Bartonella quintana* as causative agent confirming the diagnosis of bacillary angiomatosis with bacteremia. Treatment with doxycycline, initially in combination with gentamicin, led to complete resolution of the lesions. This case shows the importance of considering bacillary angiomatosis as a rare differential diagnosis of angiomatous lesions in the immunocompromised patient, particularly in chronic lymphocytic leukemia and following lymphocyte depleting treatments as alemtuzumab.

## 1. Introduction

Bacillary angiomatosis is a rare vasculoproliferative disorder due to *Bartonella henselae* or *Bartonella quintana*. The disease usually manifests as cutaneous angioma-like tumour. Lesions may be solitary or multiple and dissemination to visceral organs can occur. Bone lesions and subcutaneous masses are associated with *B. quintana,* whereas peliosis hepatis and lymph node lesions are associated with *B. henselae *[1]. Cases were usually described in HIV infected individuals. Although less common, the infection was reported in immunocompetent patients and in otherwise immunocompromised conditions such as solid organ transplantation and oncology patients, particularly in chronic lymphocytic leukemia [2, 3]. Because of its potentially life-threatening course, early diagnosis and adequate treatment are crucial. To our knowledge, we describe the first case of bacillary angiomatosis and bacteremia due to *B. quintana* in a patient with chronic lymphocytic leukemia. 

## 2. Case Presentation

A 63-year-old man was admitted for evaluation of multiple nonpruritic skin lesions that had been present for 1 month on his arms, legs, trunk, and face. His medical history was significant for chronic lymphocytic leukemia with longstanding profound neutropenia, anemia, and thrombocytopenia. He had previously been treated with chlorambucil and prednisone, as well as cladribine, rituximab, and bendamustine. At the time of presentation, he had been receiving alemtuzumab for 4 months. Current medication included prophylaxis with trimethoprim sulfamethoxazole as well as valacyclovir, and treatment with voriconazole for probable invasive pulmonary aspergillosis diagnosed during a previous febrile neutropenic episode. There had been several other episodes of neutropenic fever without specific infectious focus.

**Figure 1:**
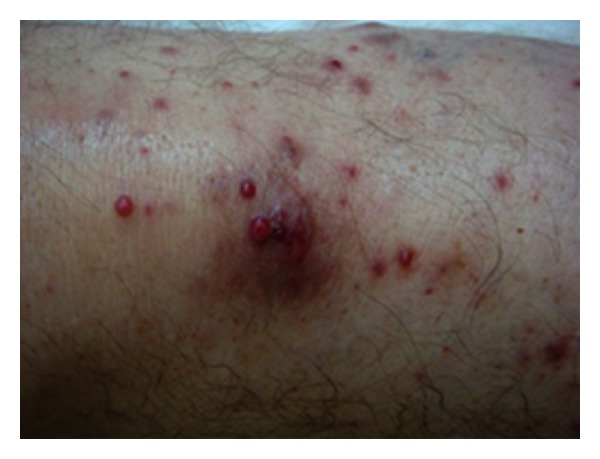
Multiple vascular nodules and papules.

**Figure 2:**
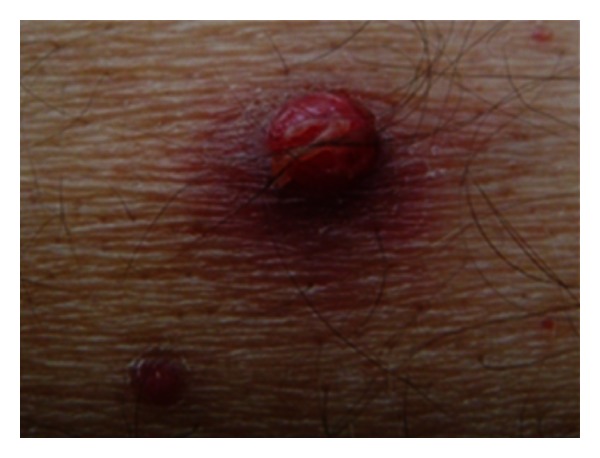
Angioma-like lesions with surrounding erythematous halo.

Physical examination showed multiple nontender cutaneous papules and nodules, resembling angiomatous lesions up to 10 mm in diameter (Figure 1). Some were crusted, and some surrounded by an erythematous halo (Figure 2). On the right forearm, there were signs of superinfection. Fever up to 39°C was noted.

The white blood count was 2600/*μ*L (absolute neutrophil count 2500/*μ*L, lymphocytes 70/*μ*L, CD4 cells 10/*μ*L, 12%, eosinophils 0/*μ*L, monocytes 100/*μ*L), CRP was 117 mg/L, and chemistry panel and urine analysis were otherwise unremarkable. Computertomography revealed known hilomediastinal and axillary lymphadenopathy, unchanged splenomegaly without focal lesions, and minimal residual pulmonary infiltrates and cavernation from the formerly diagnosed aspergillosis.

**Figure 3:**
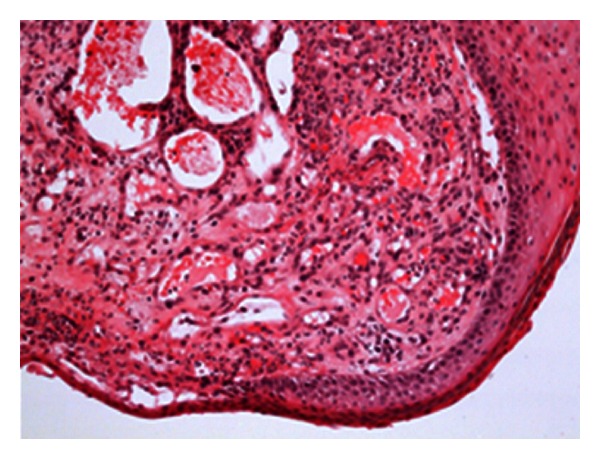
Polypoid granuloma pyogenicum-like lesion with ectatic, lobular vascular proliferation (hematoxylin-eosinstaining, 10×).

**Figure 4:**
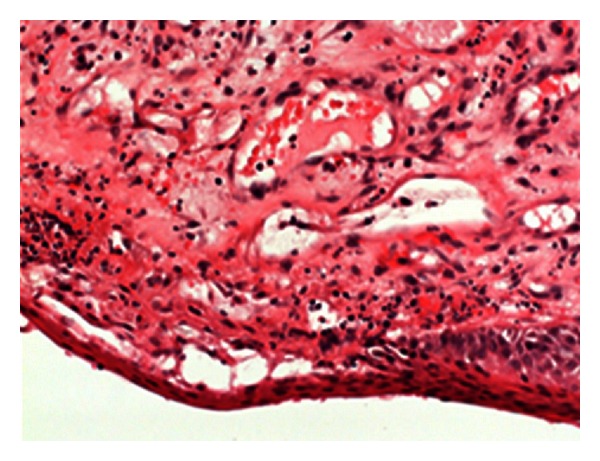
Mixed-cell infiltrates, predominantly granulocytes (hematoxylin-eosinstaining, 40×).

Biopsy of the skin revealed features of pyogenic granuloma with lobular proliferation of small vessels and mixed cell, predominantly neutrophilic inflammation (Figures 3 and 4). No microorganisms were detected on special stains as Warthin-Starry, Giemsa, Gram, or Grocott. There were no morphological signs of Kaposi sarcoma or of a vascular tumor such as hemangioendothelioma.

**Figure 5:**
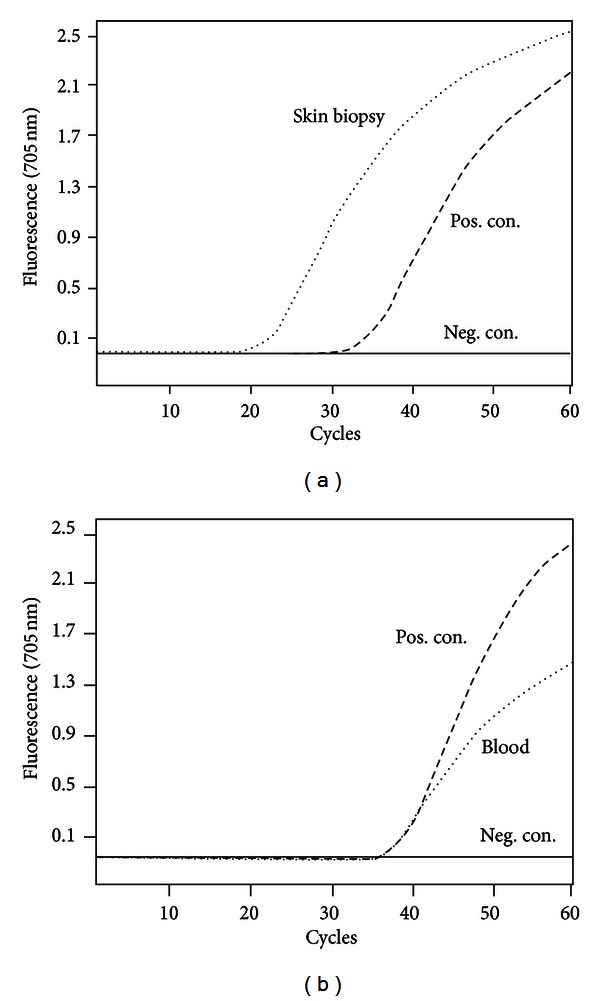
*Bartonella quintana* specific RT-PCR analysis of DNA extracts of skin biopsy (a) and blood (b).

Routine culture of skin and blood specimens showed no bacterial growth. However, RT-PCR for *B. quintana* [4, 5] was positive in a skin biopsy as well as from a blood specimen (Figure 5), confirming bacillary angiomatosis with bacteremia. To rule out endocarditis, echocardiography was performed, which showed no relevant valvulopathies or vegetations. 

We started antibiotic treatment with doxycycline and gentamicin. In addition, amoxicillin/clavulanate for superinfection of the right forearm was administered for 10 days. Gentamicin was stopped after two weeks, followed by doxycycline of 6 months duration. The patient defervesced promptly and all the lesions resolved within few weeks.

## 3. Discussion

The causative organism of bacillary angiomatosis in different case series was found to be *B. henselae* in 28% and 53%, respectively, and *B. quintana* in 64% and 47%, respectively [6, 7]. *B. quintana* infection in bacillary angiomatosis is associated with homelessness, low socioeconomic status and exposure to lice [7]. *B. quintana* was first recognized as an etiological agent of trench fever, a recurrent fever transmitted by body lice occurring in troops during World War I, whereas cat scratch disease is mainly attributed to *B. henselae. B. quintana *and *B. henselae *have worldwide distribution. Other clinical features of* B. quintana *are chronic bacteremia, endocarditis, and lymphadenopathy. Relapses of trench fever can occur many years after the initial illness or the patients may be bacteremic but have no clinical signs, as was noted in outbreaks of urban trench fever among homeless people [8]. Prolonged bacteremias in patients with *B. quintana* infections possibly contribute to the development of endocarditis and bacillary angiomatosis [9]. In contrast to *B. henselae*, *B. quintana* can cause endocarditis in previously intact heart valves. Our patient was a retiree originating from Mazedonia who had lived in Switzerland for 33 years working as a bricklayer. There was no history of cat exposure, homelessness, pediculosis, or alcoholism in our patient. Unfortunately we could not establish if it was a relapse of a previous disease or if the relapsing neutropenic fever episodes were also due to *Bartonella*. Because of prolonged bacteremia echocardiographic imaging was performed revealing no valvulopathies or signs of endocarditis.

Chronic lymphocytic leukemia appears to be a particular risk for acquiring bacillary angiomatosis due to a T-cell dysfunction similar to that of HIV patients. The depletion of CD4 cells in our patient was similar to that observed in AIDS patients, in which bacillary angiomatosis was first described in the 80s of the past century. We found five case reports of bacillary angiomatosis occurring in chronic lymphocytic leukemia patients [3, 10–13]. Most cases were diagnosed by histology, and only in one case *B. henselae *was identified as causative agent by PCR in a cutaneous specimen [12]. To our knowledge this is the first description of bacillary angiomatosis due to *B. quintana* in a patient with chronic lymphocytic leukemia. Furthermore, administration of alemtuzumab, a monoclonal anti CD52 antibody causing lymphocyte depletion, might have contributed to the particular susceptibility.

As in our case, the diagnosis of bacillary angiomatosis can be confirmed by detection of *Bartonella species* DNA in biopsy extracts by PCR. Identification of *Bartonella* by culturing is fastidious and not sensitive. Although detection of the organism in conventional cultures as performed in our patient is possible, the sensitivity could have been improved by subculture, special media, and prolonged incubation [6]. Serology may be unreliable in immunocompromised patients due to lack of antibody response [14]. Histopathology can help establishing diagnosis of cutaneous bacillary angiomatosis.

In vitro and in vivo activities of antibiotics against *Bartonella species *often differ. In epidemiological investigation trimethoprim sulfamethoxazole, ciprofloxacin, penicillins, and cephalosporins were not protective against *Bartonella* infections whereas doxycycline, tetracycline, rifampin or a macrolide were [7]. Gentamicin and rifampin were the only agents found to be bactericidal against *B. quintana* [15]. Erythromycin or doxycycline for the duration of 3 months are the drugs of choice for the treatment of bacillary angiomatosis. We preferred an initial combination of doxycycline and gentamicin because of bacteremia, as outlined in treatment recommendations for trench fever, chronic bacteremia, or endocarditis [16]. Based on the increased risk of relapse by ongoing immunosuppression in our patient, the following treatment period with doxycycline was extended to 6 months.

In conclusion, bacillary angiomatosis should be considered a differential diagnosis in immunosuppressed patients presenting with cutaneous angioma-like lesions. Particular attention should be addressed to conditions such as chronic lymphocytic leukemia and T-cell depleting therapies.
